# Elimination of HPV-associated cancers: universal HPV vaccination and the role of anal screening – a meeting report

**DOI:** 10.1186/s12919-026-00393-1

**Published:** 2026-09-22

**Authors:** F. Ricardo Burdier, Laia Bruni, Anna R. Giuliano, Nelly R. Mugo, Kirsty Roy, Joel M. Palefsky, Ann N. Burchell, Philip Roelandt, Marc Baay, Irene Man, Iacopo Baussano, Dur-e-Nayab Waheed, Alex Vorsters

**Affiliations:** 1 Centre for the Evaluation of Vaccination, Vaccine and Infectious Disease Institute, University of Antwerp, Antwerp, Belgium; 2 Unit of Infections and Cancer (UNIC-I), Cancer Epidemiology Research Programme, Catalan Institute of Oncology (ICO)–IDIBELL, L’Hospitalet de Llobregat, Barcelona, Spain; 3 Center for Immunization and Infection Research in Cancer (CIIRC), Moffitt Cancer Center, Tampa, FL USA; 4 Kenya Medical Research Institute (KEMRI), Center for Clinical Research, Nairobi, Kenya; 5 Department of Global Health, University of Washington, Seattle, WA USA; 6 Public Health Scotland, Glasgow, Scotland, UK; 7 Department of Medicine, Division of Infectious Diseases, University of California San Francisco (UCSF), San Francisco, CA USA; 8 MAP Centre for Urban Health Solutions, Li Ka Shing Knowledge Institute, St. Michael’s Hospital, Unity Health Toronto, Toronto, ON Canada; 9 Department of Family and Community Medicine, Temerty Faculty of Medicine, University of Toronto, Toronto, ON Canada; 10 Department of Gastroenterology and Hepatology, University Hospitals Leuven (UZ Leuven), Louvain, Belgium; 11 Translational Research in Gastrointestinal Diseases (TARGID), Department of Chronic Diseases and Metabolism, KU Leuven, Louvain, Belgium; 12 P95 Clinical & Epidemiology Services, Louvain, Belgium; 13 Early Detection, Prevention, and Infections Branch, International Agency for Research On Cancer (IARC/WHO), Lyon, France; 14 Centre for the Evaluation of Vaccination, Vaccine and Infectious Disease Institute, University of Antwerp, Drie Eikenstraat 663, 2650 Edegem, Belgium

**Keywords:** Human papillomavirus, Anal cancer screening, Cervical cancer screening, HPV vaccination, Males

## Abstract

The HPV Prevention and Control Board convened its 18th technical meeting in Antwerp, Belgium (June 2025) to review the evidence on universal HPV vaccination and the role of anal cancer screening in the elimination of HPV-associated cancers. While HPV prevention has historically focused on cervical cancer in women, growing evidence supports extending both primary and secondary prevention to male cohorts and non-cervical anatomical sites. HPV causes approximately 148,072 cancer cases in men annually in the world — likely a substantial undercount — affecting primarily the oropharynx, anus, and penis. Men's lower seroconversion rates after natural HPV infection and sustained HPV prevalence across age groups makes vaccination an essential tool to prevent HPV-related disease in this population. Community-randomised trial evidence confirms that universal vaccination delivers stronger herd protection than girls-only strategies and may accelerate elimination of the most oncogenic types within 15–30 years. While modelling analyses identify high coverage in girls as the most efficient strategy for cervical cancer elimination, universal vaccination is justified on broader public health grounds, including equity, programme resilience, reduced vaccine hesitancy, and prevention of non-cervical cancers. Country experiences from Scotland and Cameroon highlight persistent equity gaps and the critical role of community engagement. Given the direct epidemiological link between HPV infection in males — particularly gay, bisexual, and other men who have sex with men and immunocompromised populations, such as people living with HIV — and anal cancer risk, the meeting identified anal cancer screening as the most advanced non-cervical secondary prevention strategy warranting immediate attention. The ANCHOR trial demonstrated that treating anal high-grade squamous intraepithelial lesions reduces anal cancer incidence by 57%. Despite the publication of the first evidence-based U.S. Health and Human Services and International Anal Neoplasia Society guidelines in 2024, implementation remains constrained by high-resolution anoscopy capacity, workforce shortages, stigma, and limited evidence on optimal screening algorithms. The meeting concluded that integrating universal vaccination with targeted anal cancer screening — supported by equitable access, biomarker development, and living guidelines that evolve with emerging evidence — is essential to reducing HPV-related cancer burden in both women and men.

## Introduction

The HPV Prevention and Control Board, established in 2015, is an independent, international and multidisciplinary group of experts providing evidence-based guidance on the implementation and sustainability of HPV prevention and control programmes. The Board contributes by organising two meetings every year: a technical meeting addressing topics such as vaccine characteristics, vaccine safety, screening technologies, treatment strategies, the role of healthcare providers, and response to vaccine misinformation [1–5]; and a country meeting, structured around strengths, weaknesses, opportunities, and threats (SWOT) analysis of a country or region [6–8].

While in most regions the highest burden of disease from HPV occurs in the cervix, with female cohorts as the primary target, growing evidence supports extending primary prevention to male cohorts and secondary prevention to other HPV-associated anatomical sites, notably the anal canal. The HPV Board therefore, convened a technical meeting to review the evidence on including males as a secondary target cohort and its potential contribution to the elimination of HPV-related cancers.

This report summarises the 18th meeting of the HPV Prevention and Control Board (June 2025), which focused on universal HPV vaccination and the role of anal cancer screening in the elimination of HPV-associated cancers. The meeting was organised around four thematic areas. The first addressed the epidemiology of HPV-related disease in females and males, with attention to anatomical site distribution and the natural history of HPV infection in men. The second examined the safety, immunogenicity, and real-world effectiveness of HPV vaccination in male cohorts, including evidence of direct and indirect (herd) protection. The third considered implementation, drawing on historical transitions in other vaccination programmes (hepatitis B), vaccine supply forecasting, cost-effectiveness, and country experiences among both adopters and non-adopters of universal vaccination. The fourth focused on the current landscape of anal cancer screening, including available guidelines, integration with existing programmes, and barriers to equitable implementation.

### Gender-neutral versus routine versus universal vaccination

In the current global political climate, terminology may influence how programmes are perceived and adopted. The use of the term “*Gender-neutral vaccination*” may not be most appropriate as it places emphasis on gender; “*routine vaccination*” implies a systematic approach, as distinct from campaigns targeting specific groups or outbreaks; Ultimately “*universal vaccination*” implies immunisation without discrimination. Although an age qualifier could be added (e.g. “*universal adolescent/childhood vaccination*”), this risks confusion given that HPV vaccination is increasingly recommended from the age of 9, outside the adolescent window. Throughout this report the term *universal (HPV) vaccination* is used where possible.

## Burden of HPV-related disease

### Global distribution of HPV-related disease

Cervical cancer remains the most incident and prevalent HPV-related malignancy in women, with HPV identified as a necessary cause in most cases. In 2022, over 600,000 new cases and more than 300,000 deaths were attributed to cervical cancer globally. The highest incidence and mortality rates are concentrated in Latin America, the Caribbean, Sub-Saharan Africa, Southeast Asia, and Eastern Europe [9]. Age-adjusted incidence and mortality are lower in high-income countries (HICs), while the absolute case numbers are highest in middle-income countries due to larger populations; China and India alone account for over 40% of global cases. Incidence has declined in many regions over recent decades, but is stagnating or rising in Sub-Saharan Africa and Eastern Europe.

HPV is also etiologically linked to anal, vulvar, vaginal, penile, and oropharyngeal cancers [10–12]. The HPV-attributable fraction varies by site: 80–90% of anal cancers, ~ 80% for vaginal cancer, ~ 50% for penile cancer, ~ 25% for vulvar cancer, and ~ 30% globally for oropharyngeal cancer, with strong regional variation [12]. In the U.S. and Northern Europe, up to 80% of oropharyngeal cancers are HPV-related, compared with less than 10% in other regions. HPV plays a minimal role (< 5%) in oral cavity and laryngeal cancers.

HPV 16 is the predominant genotype across nearly all HPV-associated cancers, including cervical squamous cell carcinoma, oropharyngeal cancer, and other anogenital cancers, with limited variation between sexes. HPV 16, 18 and 45 are more frequently implicated in adenocarcinomas.

While anal cancer incidence is highest among gay, bisexual, and other men who have sex with men (GBMSM) and people living with HIV (PLWH), immunocompetent women represent the largest affected group in absolute numbers. Oropharyngeal cancer incidence is rising in regions where tobacco and alcohol-related cancers are declining, consistent with HPV as the principal driver of head and neck carcinogenesis in these regions [13, 14].

### Burden of HPV-related disease in men

HPV contributes substantially to disease burden in males, driving genital warts, recurrent respiratory papillomatosis (RRP), and cancers of the anus, penis, and oropharynx. Globally, approximately 148,072 new HPV-related cancers occur in men each year, with age-standardised incidence rates per 100,000 highest in North America (6.0), Europe (5.5) and Oceania (5.3) [15]. Oropharyngeal cancer is the most common HPV-associated malignancy in men: incidence among males in HICs has more than doubled over the past 15 years, now exceeding that of cervical cancer in women, and men are five times more likely to be diagnosed than women [16, 17].

Men have a lower seroconversion rate than women following natural genital HPV infection, suggesting prolonged susceptibility [18, 19]. Unlike in women, in whom HPV prevalence declines with age, oral and genital HPV prevalence remains relatively constant across age groups [20, 21].

Anal HPV infection is common in males, particularly among GBMSM and immunocompromised individuals.

Epidemiological data from 64 studies covering approximately 29,900 men indicate wide variability in anal HR-HPV (high-risk HPV) prevalence by risk group [22]: 74.3% among GBMSM living with HIV, 41.2% among HIV-negative GBMSM, and 6.9% in HIV-negative men who have sex with women [23, 24]. GBMSM living with HIV experience the highest incidence, and reduced immune control leads to longer periods of detectable infection and a greater likelihood of persistence [25–27]. High-grade squamous intraepithelial lesion (HSIL) is established as a direct precursor to anal cancer [28], and prevention in the highest-risk groups requires both early vaccination and targeted screening — evidence reviewed in detail in Sects. 7 and 8.

### Global status of primary and secondary prevention

By 2025, 149 out of 194 countries (77%) had introduced the HPV vaccine into their national immunisation schedules [29]. The proportion of countries within a region offering HPV vaccination is highest in Oceania (94%) and Latin America and the Caribbean (91%) and lowest in Sub-Saharan Africa (60%) and Northern Africa and Western Asia (52%); 55% of girls worldwide still live in countries without an HPV vaccination programme.

Global HPV vaccine coverage remains below the World Health Organization (WHO) 2030 target of 90%, but adoption of the WHO-recommended single-dose schedule (December 2022) has accelerated scale-up. By 2023, 75 countries (50% of those with HPV vaccination programmes) had adopted single-dose regimens; approximately 28 million girls and 6 million boys aged 9–14 received a first dose that year, with 47% of girls and 17% of boys vaccinated under the single-dose approach. However, only 16 countries achieved > 90% first-dose national coverage in 2023, and average first-dose coverage increased modestly from 55% (2021) to 62% (2023) with final-dose coverage rising from 44 to 52% [29].

The global cervical cancer screening landscape remains fragmented [30]. In 2022, estimated five-year global screening coverage among women aged 30–49 was just 32% [31]. HPV-based screening reached only 7%. Coverage varies markedly by region: 64% in Latin America and the Caribbean but under 15% in most low-income regions such as Sub-Saharan Africa and Central Asia, and less than 1% of women in many low- and lower-middle-income countries have ever undergone HPV testing [31].

HPV-based primary screening is recommended in 83 of 161 countries with guidelines. However, fewer than half operate organised, invitation-based programmes. Lastly, self-sampling is formally recommended in only 42% of these. Closing these gaps, alongside expanded vaccination, is essential to achieving cervical cancer elimination.

### Discussion

Two points from the meeting discussion warrant emphasis. First, while most studies place the HPV-attributable fraction of cervical cancer at or near 100%, studies reporting 5–10% HPV-negative cases reflect variation in test sensitivity and methodology — a distinction that matters in contexts with high misinformation and scepticism towards HPV such as Japan, where clear communication is essential to countering misinformation. Second, the frequently cited estimate of 148,072 annual HPV-related cancer cases in men likely represents a substantial underestimation; conservative re-estimates suggest approximately 180,000 cases annually. A consensus on a more robust estimate would objectively strengthen the evidence base for universal vaccination policy in both sexes.

## HPV vaccination in male cohorts: safety, efficacy, impact and equity

### HPV vaccination in male cohorts: safety and immunogenicity

Long-term follow-up in men aged 16–26 have demonstrated strong, durable efficacy of quadrivalent and nonavalent vaccines, with no new cases of external genital disease such as genital warts or high-grade anal intraepithelial neoplasia (AIN) related to vaccine types [32, 33] with antibody responses persisting for at least 10 years, and only one new case of AIN reported [34].

Population-level benefits have also been documented. Countries with universal programmes, such as Australia, Canada and the UK have seen rapid declines in genital warts and oral HPV prevalence, with Australia reporting reductions in juvenile-onset RRP and oral HPV infection in young adults since vaccine introduction [35].

Universal vaccination adds direct protection for males to herd effects, strengthens programmatic resilience to disruptions, and accelerates the elimination. Modelling suggest that high-risk HPV elimination could be feasible within 30 years if global coverage exceeds 75% [36].

### Herd protection inferred by vaccinating male cohorts

A series of randomised controlled trials in Finland provides strong evidence on the effectiveness of universal vaccination policies [37–39]. Between 2007 and 2014, a community-randomised trial across 33 Finnish communities enrolled over 80,000 adolescents, randomised to universal HPV16/18 vaccination, girls-only HPV16/18 vaccination or control receiving hepatitis B virus (HBV) vaccination, with overall coverage of 52% in girls and 29% in boys.

The findings consistently favoured universal vaccination [40]. In communities where both boys and girls were vaccinated, HPV16/18 prevalence was significantly reduced not only among vaccinated but also among unvaccinated females, demonstrating a robust herd effect. This effect was stronger in the universal arms (p for trend 0.0005 compared to controls) than in girls-only arms (p for trend 0.092 compared to controls) [41]. Prevalence of other high-risk types (HPV18, 31, 33, 35) was also markedly lower in the universal vaccination group, suggesting broader ecological effects. By contrast, girls-only and lower-coverage arms offered limited herd protection, leaving unvaccinated and marginalised women disproportionately affected.

Taken together, these findings suggest that even moderate-coverage universal HPV vaccination can deliver substantial public health benefits within five years, including strong herd protection. Modelling and ecological observations further suggest that elimination of the most oncogenic types (HPV16 and 18) may be feasible within 15–30 years if universal vaccination strategies are widely adopted.

### Discussion

Data on male HPV-related cancers in LMICs remain scarce compared to cervical cancer, although local evidence indicates a rise in penile cancer and in oropharyngeal cancer approaching incidence levels reported in HIC. Because sustaining 90% coverage in girls alone is difficult, universal vaccination can also accelerate reductions in cervical cancer. In resource-limited settings, the relevant objectives — elimination, resource efficiency, and equity — may point in different directions. High coverage in girls generates herd effects, but the Finnish community-randomised trial showed that these effects were significantly stronger under universal strategies. Finally, girls-only vaccination does not address the HPV reservoir in boys, whereas universal vaccination does, so, for accelerated elimination, both sexes must be vaccinated. In LMICs, however, resource constraints may preclude this.

## HPV vaccine availability, pricing and supply dynamics: informed decision-making for policy & procurement

### Where are we now? WHO’s vision

In response to the WHO’s Cervical Cancer Elimination Initiative and the adoption of a single-dose schedule for girls aged 9–14, support from Gavi, the Vaccine Alliance, has driven broader programmatic uptake, including catch-up and male vaccination efforts in LMICs, although the latter is not Gavi-supported. New manufacturers and ongoing technology transfers, particularly in China, are reshaping supply dynamics. Five vaccines from four manufacturers currently hold WHO prequalification (Table 1).

**Table 1:** Characteristics and composition of WHO prequalified HPV vaccines

Vaccine	Bivalent			Quadrivalent	Nonavalent
Trade name	Cecolin™	Cervarix™	Walrinvax™	Gardasil™	Gardasil-9™
Manufacturer	Xiamen Innovax Biotech Co. Ltd	GSK SA	Walvax Biotech Co. Ltd	Merck	Merck
Date WHO prequalification	14/Oct/2021	8/Jul/2009	2/Aug/2024	20/May/2009	9/Feb/2018
Antigens	16,18	16,18	16,18	6,11,16,18	6,11,16,18,31,33,45,52,58
Adjuvant	Aluminium hydroxide	AS04	Aluminium phosphate	Aluminium hydroxy-phosphate sulfate	Aluminium hydroxy-phosphate sulfate
Expression system	*Escherichia coli*	Baculovirus	*Pichia Pistoris*	*Saccharomyces cerevisiae*	*Saccharomyces cerevisiae*

Adapted from [42]. ASO4 – aluminium hydroxide and 3-deacetylated monophosphoryl lipid A

### Pipeline of vaccines

In India, a domestically produced HPV vaccine with a male indication was licensed in 2022 on the basis of immunobridging to Gardasil; although not yet WHO-prequalified, the manufacturer has committed to scaling production to 100 million doses annually. In China, development is advancing rapidly, with ten higher-valency candidates in clinical stages, including one in Biologics License Application filing and several in Phase 3 trials targeting both sexes (Table 2).

**Table 2:** HPV vaccine R&D pipeline in China^†^

Company	Vaccine	Expression system	Stage
CNBG/CDIBP	HPV-4 (6,11,16,18)	Yeast (*H. polymorpha*)	cBLA
Innovax	HPV-9 (6,11,16,18,31,33,45, 52,58)	*E. coli*	cBLA in females, ph3 in males in preparation*
Health Guard	HPV-3 (16,18,58)	*E. coli*	Ph3 females finished
Bovax	HPV-9 (6,11,16,18,31,33,45, 52,58)	Yeast (*H. polymorpha*)	Ph3 females + males ongoing
Health Guard	HPV-9 (6,11,16,18,31,33,45, 52,58)	*E. coli*	Ph3 females + males ongoing
RecBio	HPV-9 (6,11,16,18,31,33,45, 52,58)	Yeast (*H. polymorpha*)	Ph3 females ongoing
CNBG/CDIBP	HPV-11 (6,11,16,18,31,33, 45,52,58,59,68)	Yeast (*H. polymorpha*)	Ph3 females ongoing
SCT	HPV-14 (6,11,16,18,31,33, 35,39,45,51,52,56,58,59)	Insect cells (baculovirus)	Ph3 females ongoing
Zerun/Walvax	HPV-9 (6,11,16,18,31,33,45, 52,58)	Yeast (*P. pastoris*)	Ph2 females finished
CNBG/SIBP	HPV-4 (16,18,52,58)	Yeast (*P. pastoris*)	IND, ph 1/2
Immune Path	HPV-9 (6,11,16,18,31,33,45, 52,58)	Yeast (*P. pastoris*)	IND, ph 1/2
Bovax	HPV-15 (6,11,16,18,31,33, 35,39,45,51,52,56,58,59,68)	Yeast (*H. polymorpha*)	IND, ph 1/2
Chengda (Health Guard)	HPV-15 (6,11,16,18,31,33, 35,39,45,51,52,56,58,59,68)	*E. coli*	IND, ph 1/2

Source: NMPA data; clinicaltrial.gov. Enterprise official website information; updated in Jan. 2025; cBLA: Confirmation for Biologics License Application (BLA); *: Immunogenicity study only- not clinical efficacy; ^†^ This table takes into consideration only products in the pipeline submitted by Chinese companies; thus, other regions are not included

### Where are we now? UNICEF’s vision

Between 2011 and 2016, the global HPV vaccination effort was shaped by the opening of the Gavi funding window, a two-dose schedule recommendation, and UNICEF supply agreements with two suppliers, although demand remained low due to programmatic challenges. From 2017 to 2024, the HPV revitalisation programme and the shift to a single-dose schedule led to a demand surge that outpaced supply, prompting UNICEF to expand its supplier base to three manufacturers; supply capacity improved by 2025.

The supply–demand dynamic is projected to balance in the mid to long term, with supply potentially exceeding demand. Despite these advances, several persistent barriers affect country-level decision-making, including affordability, sustainability, procurement strategies and the accuracy of long-term demand forecasts. These challenges are best addressed through pooled procurement and multi-year commitments to ensure consistent and equitable access.

### Discussion

The current supply situation arguably creates an opportunity for universal vaccination, however, the Gavi eligibility rule — whereby vaccines procured with Gavi support must be administered to girls, and any doses given to boys require the government to reimburse Gavi at the full vaccine cost— still reflects the earlier supply shortage period. WHO recommendations are grounded in health impact rather than market dynamics: modelling shows high-coverage girls-only vaccination, as achieved in many LMICs, is sufficient for cervical cancer elimination and adding boys does not reliably increase coverage. In low-resource settings, programme sustainability is the central challenge. Evidence from Cameroon suggests that universal HPV vaccination can, in some contexts, enhance both acceptability and programme sustainability. However, this is not universal — in many settings, expanding vaccination to boys doubles the doses required without meaningfully improving sustainability, whereas achieving high coverage in girls does. Further implementation research is paramount to understand what drives sustainable HPV vaccination programmes. As supply grows, UNICEF is shifting its focus from routine girls-only vaccination to multi-age cohorts; universal vaccination will be considered only once these cohorts are covered.

Merck/MSD, which supplies 150 national immunisation programmes, has invested USD 2 billion over the past six years to expand capacity for universal and adult vaccination, and has committed to meeting 100% of UNICEF’s demand over 2026–2028. Two large trials looking into the efficacy of single dose nonavalent vaccine in males (aged 16- 26) are ongoing, aimed at filling current evidence gaps. Several countries that introduced HPV vaccination later have opted for low-cost vaccines, anticipating the time when they will no longer be Gavi-eligible and will have to pay the full price for the vaccines.

Managing this evolving landscape will require careful demand forecasting. Supply is projected to stabilise at around 100 million doses annually, potentially peaking at 130 million under universal vaccination — but manufacturing scale-up is not always straightforward, not all pipeline candidates carry a male or single-dose indication, and many require additional efficacy data before market entry. Overproduction risks can be mitigated through longer-term demand commitments, while pooled procurement and multi-year agreements remain the most reliable tools for ensuring consistent and equitable access.

## From data to policy of universal vaccination programmes (HIC & LMICs)

### Historical shifts in other vaccination programmes, with a focus on Hepatitis B

Historical precedents inform the transition from girls-only to universal HPV vaccination. Early HBV immunisation strategies focused on high-risk groups — healthcare workers, people who inject drugs, sex workers, and individuals with high-risk sexual behaviour — but failed to substantially reduce HBV-related morbidity and mortality despite a safe and effective vaccine available since 1981 [43, 44]. Contributing factors include difficulty in identifying risk groups, limited public awareness, stigma and insufficient funding and infrastructure. Recognising these limitations, the public health community gradually shifted toward universal immunisation, targeting entire age cohorts — particularly newborns and infants — regardless of individual risk. Universal vaccination programmes delivered free through structured healthcare systems, such as child health clinics, achieved broader coverage and reduced the overall transmission of HBV at the population level.

In 1992, WHO recommended integrating HBV vaccine into national immunisation programmes by 1997 [45]. By 2013, 47 out of 53 countries in the WHO European Region had adopted universal HBV immunisation. The establishment of Gavi in 2000 further expanded access in the world’s poorest countries, and many African countries increased infant coverage from 40% to around 70%.

Modelling studies estimate that HBV will avert approximately 14 million deaths between 2021 and 2030, underscoring its tremendous public health impact [46]. The HBV experience demonstrates the superiority of universal vaccination over risk-based vaccination for public health outcomes: although universal programmes require vaccinating large numbers of low-risk individuals, the resulting gains in community-level impact and progress towards elimination justify the investment — a lesson directly applicable to HPV.

### Global insights on universal HPV vaccination: lessons from implementing countries

#### United Kingdom

The school-based, universal HPV vaccination programme in Scotland is a key component of the country’s national immunisation strategy. The programme was introduced for girls in 2008 and extended to include boys in 2019. The vaccine is offered free at the point of delivery to all eligible adolescents, and is routinely delivered at around 12–13 years of age, typically during the first year of secondary school.

Implementation is decentralised across 14 regional NHS Boards with national coordination. Delivery follows a structured process: local authorities provide school roll data, consent packs (including letters, forms, and educational materials) are distributed to parents and carers, and vaccinations are administered on-site at schools by healthcare teams. Alternative delivery pathways exist for students outside of mainstream education or who are absent on scheduled vaccination days, including catch-up opportunities beyond the routine school year.

Historically, vaccine uptake has been higher among female students, but targeted efforts are being made to address the gender gap. Ongoing initiatives to improve coverage include national public awareness campaigns (e.g. “Chat Sign Protect”), tailored community-specific engagement (such as with Gypsy, Roma and Traveller groups), and exploration of digital consent methods. Ongoing monitoring and evaluation remain essential, particularly in identifying and addressing both attitudinal and practical barriers affecting vaccine uptake.

Overall, the Scottish experience highlights the value of coordinated healthcare delivery, robust consent processes, and targeted outreach in achieving equitable immunisation coverage.

#### The perspective of LMICs

The burden of HPV-related cancers is greatest in LMICs, particularly among women. Most HPV vaccination efforts in LMICs target only adolescent girls, as many countries depend on Gavi, which does not support male HPV vaccination. This selective approach can exacerbate gendered vaccine hesitancy, often linked to fears of infertility, moral judgment and social stigma. Misinformation, sometimes originating from respected sources including religious leaders and politicians, further undermines trust. Studies in Sub-Saharan Africa highlight that male exclusion from vaccination programmes also excludes them from critical HPV education, reinforcing misconceptions and limiting community support [47, 48]. Universal vaccination has been recommended to improve coverage, promote equity and address misinformation.

Cultural sensitivities, limited health infrastructure and patriarchal decision-making further complicate vaccine delivery. Fear of side effects and fertility-related concerns persist across diverse populations. Comprehensive strategies, including inclusive communication, stakeholder engagement, and community-led education, are vital to overcoming these barriers.

#### Cameroon

In Cameroon, where HPV-related burden is a major public health concern, early HPV vaccination pilot programmes (2010–2016) demonstrated strong community engagement, with 74% of girls in Foumban and Edéa completing both vaccine doses [49]. This promising start led to a nationwide rollout in 2020, targeting 9-year-old girls with the Gardasil-4 vaccine.

The programme faced significant setbacks. Launched during the COVID-19 pandemic, it encountered strong resistance from religious leaders and some healthcare professionals, leading to the cancellation of the school-based strategy and poor initial coverage, particularly in urban areas like Douala, Yaoundé, and Bafoussam. The Catholic Church banned the vaccine in its facilities, which represent 30% of health infrastructure, while contradictory messages from public health experts and the spread of misinformation further weakened uptake.

Two years post-launch, 84% of health facilities were not offering HPV vaccination. In response, the strategy shifted to a single-dose schedule, extended to include boys, and used periodic intensification of routine immunisation (PIRI). The revamped programme main pillars were: 1) A renewed focus on community partnerships by improving the management of related stakeholders such as religious bodies, which then became spokespersons for HPV vaccination 2) Socio-cultural dialogue by co-creating and deploying communication activities by and closer to the community, expanding to youth centres, places of worship and chiefdoms and 3) Service diversification by also offering HPV vaccination beyond schools to youth centres. All regions improved, with four (Adamawa, East, Far North, and North) having coverages for girls over 90% and around 40% for boys. PIRI improved girls' vaccination in 70% of regions while universal single-dose vaccination improved coverage in 30% of regions [50]. Nevertheless, intensified efforts are required to achieve cervical cancer elimination targets by 2030, particularly in screening and care, where data availability and infrastructure remain limited.

### Discussion

In Cameroon, the transition from girls-only to universal vaccination proved a turning point: it neutralised suspicions of hidden programme motives, and coverage in girls subsequently increased — suggesting that equity framing strengthened public acceptance. It should be noted, however, that the shift to universal vaccination was implemented concurrently with PIRI and broader community engagement strategies, making it difficult to attribute coverage gains to any single intervention. Disentangling the independent contribution of universal vaccination from these accompanying measures remains an important question for future implementation research. Engagement with religious stakeholders proved critical. Cameroonian authorities initiated structured dialogue with all major religious groups, most of which were ultimately persuaded of the public health value of HPV vaccination — with the Catholic Church as a notable early exception. Muslim religious leaders went further, issuing a formal white paper endorsing HPV vaccination, which contributed to high coverage in predominantly Muslim regions.

In HICs such as Scotland, barriers vary across population subgroup. In rural areas, geographical access remains a key constraint, whereas in other communities social and economic priorities reduce uptake. Administrative processes present a structural barrier: reliance on paper-based consent forms often results in forms being lost or not returned, resulting in missed vaccinations. Some areas mitigate this by telephoning parents on the day of vaccination, but this approach is resource-intensive and hard to sustain at scale. Transitioning to digital consent systems has the potential to mitigate these losses and improve efficiency. Despite these challenges, uptake is partly improved by the design of the school-based programme in Scotland, which includes repeated catch-up opportunities through secondary school. Adolescents who miss vaccination during school-based sessions are re-offered the vaccine in subsequent years helping to account for absenteeism and late consent. Individuals remain eligible up to the age of 25. Once people leave school, vaccination is no longer organised through routine invitation and relies on opportunistic, self-initiated access within healthcare settings. This shift from an actively organised programme to one that relies on individual initiative introduces an additional equity challenge. Individuals from more disadvantaged backgrounds may face greater barriers to navigating the healthcare system, increasing the risk that those underserved during the school years remain unvaccinated in adulthood.

## Implementation of universal HPV vaccination: lessons learned, feasibility and cost-effectiveness

### Has the inclusion of male cohorts impacted equity? Implementation insights from the UK as a pioneer of this policy

Scotland’s HPV vaccination programme, introduced in 2008 for girls and expanded universally to boys in 2019 — with an interim targeted programme for GBMSM via sexual health clinics from 2017 — provides one of the most developed real-world datasets on the equity implications of universal vaccination.

The rationale for vaccinating males combines direct protection against multiple HPV-related cancers (penile, anal, and oropharyngeal), especially in high-risk populations such as GBMSM who represent heightened risk, prevention of genital warts and associated psychosexual issues, herd protection for unvaccinated individuals, and gender equity in cancer prevention. Although the Joint Committee on Vaccination and Immunisation (JCVI) initially deemed universal vaccination not cost-effective, revised models incorporating long-term savings and GBMSM protection supported its implementation.

Although Scotland provides universal, school-based access to the vaccine, uptake remains uneven [51]. Overall coverage has declined over the past decade, with a persistent gap between boys and girls. Socio-economic deprivation also affects uptake, with inequalities widening over time. Among pupils in S4 (Secondary 4 is the fourth year of secondary school) in 2024/25, coverage was 75% in those living in the most deprived areas, compared with 91% in the least deprived, a gap that has increased substantially since 2019/20 when the vaccination programme was extended to include boys. These patterns are particularly pronounced among boys [51]. Lower awareness of HPV and its association with non-cervical cancers among boys may contribute to these differences, possibly reflecting the programme’s historical focus on cervical cancer prevention. Ethnic disparities are also apparent, with lower uptake among most minority groups compared to the white population, potentially compromising herd immunity within certain communities.

Regardless, when compared to the pre-vaccination cohorts, direct and indirect effects of HPV vaccination programmes are clear. Early evidence from Scotland suggests that the HPV vaccination programme is generating indirect (herd) protection. Declines in prescriptions for genital wart treatment — used as a proxy indicator of HPV transmission — have been observed in vaccinated female cohorts, and a similar downward trend among certain male cohorts who were not eligible for vaccination, suggesting early signs of herd protection.

Evidence from female cohorts further demonstrates the vaccination programme’s role in reducing health inequities [52, 53]. Among vaccinated women, rates of high-grade cervical intraepithelial neoplasia (CIN3) do not differ significantly by socio-economic status, unlike unvaccinated women, where a clear deprivation gradient persists [52]. This suggests that vaccination may attenuate the strong social patterning historically associated with cervical disease incidence.

However, lower cervical screening coverage among unvaccinated women raises concerns about a subgroup experiencing compounded vulnerability, characterised by both lack of direct vaccine protection and reduced participation in screening [54].

### Modelling the impact of universal HPV vaccination

Mathematical modelling can illuminate the projected impact of various vaccination strategies on elimination and on health inequalities. The HPV-ADVISE model [55] — a transmission-dynamic agent-based framework calibrated to six core countries and mapped to 67 LMICs and 40 HICs [56] — evaluates vaccination strategies by their efficiency (number needed to vaccinate (NNV) to prevent one cervical cancer case) and their capacity to reduce incidence and promote equity.

The analysis identified routine single-dose HPV vaccination for 9-year-old girls as the most efficient strategy. Single-dose efficacy proved comparable to a 2-dose regimen making the second dose redundant in many cases. Adding multi-age cohort vaccination of girls up to age 20, further improved impact, with NNVs of 48 to 64, whereas adding vaccination for boys offered limited additional benefit due to herd immunity conferred by high coverage in girls, with significantly higher NNVs (e.g., > 500 for boys aged 10–20).

Model projections under the current status quo revealed limited impact on cervical cancer incidence in LMICs and a widening disparity compared to HICs. If no changes are made, age-standardised incidence in LMICs is projected to be 15 times higher than in HICs by 2105. Conversely, strategies aligned with WHO’s elimination targets could substantially narrow the equality gap and accelerate elimination timelines (LMIC 1.25 higher than HIC). High universal HPV vaccination coverage would close the gap between LMICs and HICs.

Nevertheless, increasing coverage in girls and young women in these models proved far more efficient than expanding vaccination to boys, especially in contexts with limited resources. For example, increasing vaccination coverage in girls from 40 to 80% yielded up to three times the reduction in cervical cancer cases compared to initiating vaccination in boys at the same coverage level. The efficiency of strategies also varied by cervical cancer burden. In high-burden settings, prioritising female vaccination remained optimal, while in lower-burden countries, vaccinating older women became more favourable than adding boys.

To support evidence-informed decision-making for cervical cancer prevention in LMICs, the International Agency for Research on Cancer (IARC/WHO) has developed a comprehensive modelling framework. This METHIS modelling platform [57] integrates validated transmission and disease progression models (e.g., RHEA, ATLAS) with economic models drawing on global and country-specific empirical data (e.g., GLOBOCAN, DHS, UNAIDS) and curated databases such as CHRONOS (HPV prevalence) and COEUS (cervical cancer societal costs). This setup allows for tailoring models to individual LMICs while imputing missing variables based on similarity clusters ("footprints") in sexual behaviour and disease burden [58].

One key focus is defining optimal female catch-up vaccination age ranges, using the concept of dose efficiency to determine the most equitable strategy. Two perspectives are considered: A local perspective, which maximises relative gain of efficiency within a country and hence strives for equity within a country; and a global perspective, which applies a standard threshold (e.g., maximum 250 doses per case prevented) for equitable dose distribution across all countries.

Cost-effectiveness analyses conducted in six LMICs (India, Indonesia, Kenya, Nigeria, Colombia, and Eswatini) confirmed that catch-up vaccination up to age 30 could be cost-effective in many settings, and even cost-saving in India and Eswatini due to high cervical cancer treatment costs relative to gross domestic product (GDP) [59]. Extending the catch-up age to 24–30 years remained potentially cost-effective in many settings but was constrained by vaccine supply, programmatic feasibility, and local health budgets. Under ideal conditions (90% vaccine coverage, single-dose schedule), catch-up age ranges up to 18 and 30 years corresponds to 9.4% and 21.7% of the 5-year immunisation budget, respectively. Overall, expanding HPV vaccination up to age 30 would avert 9.3 million cervical cancer cases in LMICs and would require approximately 986 million vaccine doses if a single-dose regimen would be adopted.

Prioritising high coverage vaccination of girls and young women (up to 20 years) offers high impact and efficiency. Expanding catch-up age beyond this range may be justified where resources allow but must be weighed against cost and supply constraints. Integration of modelling with local data and policy realities is essential to advancing equitable cervical cancer elimination worldwide.

### Discussion

Cost-effectiveness modelling in LMICs is frequently constrained by limited availability of high-quality local costing data. Of over 130 LMICs, only approximately 20 had sufficient data quality to support robust economic modelling reducing confidence in national projections and policy recommendations. Improving model accuracy requires direct engagement with national stakeholders, collaboration with policymakers, and systematic collection of local epidemiological and costing data. Importantly, many valuable datasets exist within ministries or institutions but remain unpublished. Accessing and incorporating these local data sources substantially strengthens model calibration and policy relevance.

The WHO 90–70–90 framework is a validated pathway to cervical cancer elimination. However, it is not the only viable strategy. Countries may adapt the framework to align with local infrastructure, fiscal capacity, and demographic realities. Approaches such as “one vaccination–one screening” may provide pragmatic alternatives, particularly in large or resource-constrained settings. While cost-effectiveness modelling consistently positions female-targeted vaccination as the most efficient strategy — given the higher NNVs associated with including males — this framing has limitations. Cost-effectiveness is necessary but not sufficient for policy decision-making. When equity, acceptability, and long-term elimination goals are incorporated, the policy implications differ. Universal vaccination strategies, despite their lower efficiency in preventing cervical cancer cases per dose, play a critical role in reducing the projected incidence gap between LMICs and HICs. It is therefore important to distinguish between i) efficiency in reducing cervical cancer alone, and ii) broader public health objectives, including elimination of all HPV related cancers, equity across populations, and programme resilience. This distinction is central to policy decisions. Achieving elimination of HPV-related cancers requires strategies that address burden across all affected populations, not only those where the cost-per-outcome ratio is most favourable. A further consideration is that most modelling exercises use cervical cancer as the primary endpoint. While inclusion of other HPV-related cancers does not substantially alter conclusions in current models, this should be interpreted with caution. It likely reflects limitations in surveillance and data availability rather than the true absence of benefit. As screening and data systems for non-cervical HPV-related cancers improve, the relative value of universal vaccination is likely to be reassessed.

## Current landscape of anal cancer screening

### HPV-related burden of disease in the anal canal: similarities and differences with the cervix

Although anal cancer incidence is relatively low in the general population (1–2 cases/100,000), it is markedly elevated in specific groups — GBMSM, PLWH, women with HPV-related gynaecological conditions, transplant recipients, and patients undergoing immunosuppressive therapy [60, 61] — driven primarily by increased susceptibility to persistent HPV16 infection, the dominant oncogenic HPV type associated with anal squamous cell carcinoma (SCC).

Notably, anal SCC cases are not evenly distributed worldwide, with regional differences reflecting variations in HIV prevalence, healthcare infrastructure (possibly also affecting quality of cancer reporting) and antiretroviral (ART) access [62, 63].

HPV16 causes the vast majority of cases across risk groups [64], making its prevalence a critical biomarker for assessing cancer risk. A pooled analysis involving 64 studies and over 29,000 men [22], and 26 studies covering 11,000 women [65], showed striking differences in HPV16 prevalence by gender, HIV status, and sexual behaviour with GBMSM — especially those living with HIV — consistently demonstrating the highest prevalence. A particularly high prevalence was observed among younger GBMSM (15–25 years), suggesting early acquisition.

Among women, shifts in HPV16 prevalence from the cervix to the anus with increasing age has been documented, particularly in those with prior HPV-related cervical pathology [65]. Longitudinal data from 34 studies covering 16,000 individuals further characterised natural history of anal HPV16: persistent infection emerges as the critical driver of progression to high-grade lesions and cancer, with incident infection associated with recent number of sexual partners (adjusted hazard ratio [aHR] = 1.22 for GBMSM, 2.58 for women), and HIV status (aHR = 1.42 for GBMSM, 1.90 for women) [27]. Together, these findings underscore the importance of longitudinal monitoring and risk stratification in ageing populations with a history of HPV-associated disease.

Prophylactic HPV vaccination is effective at the anal site [33]. Multiple studies confirm significant reductions in HPV16 incidence and related disease, particularly when administered prior to exposure [66, 67]. Effectiveness correlates with naivety to anal HPV infection (younger age, fewer lifetime sexual partners) and diminishes with increasing age and prior exposure.

### Prevention of HPV-related disease of the anus: primary and secondary prevention

Primary prevention is primarily focused on HPV vaccination. Studies have demonstrated that the quadrivalent vaccine is highly effective in preventing persistent anal HSIL and high-risk HPV infections [32, 33]. Long-term follow-up data reveal sustained protection extending up to 15 years, with similar efficacy and safety profiles in males as observed in females [34]. However, as in the cervix, a critical caveat is that vaccination must occur prior to HPV exposure, ideally before the onset of sexual activity. The vaccine's efficacy declines sharply in individuals with prior exposure to the targeted HPV types. Data from younger populations show that by age 20, nearly half of GBMSM living with HIV had already been exposed to vaccine-covered HPV types, underscoring the urgency of early vaccination [68]. Nevertheless, evidence suggests that in individuals naive to the relevant types, especially those under 35, vaccination significantly reduces incidence of high-grade disease. Hence, maximising vaccine impact requires timely administration within the recommended age range.

Secondary prevention involves screening for and treating HSIL to prevent progression to invasive anal cancer. The pivotal ANCHOR study provided strong evidence supporting secondary prevention of anal cancer [28]. In a randomised controlled trial involving over 10,000 individuals with HIV, treatment of biopsy-proven HSIL through office-based electrocautery significantly reduced progression to anal cancer by 57% compared to active monitoring. Notably, 53% of men, 47% of women, and 67% of transgender individuals screened were diagnosed with HSIL, highlighting the high prevalence of pre-cancerous lesions in these populations.

Although the ANCHOR study was not intended to determine the ideal treatment option (electrocautery, imiquimod and TCA were used), the study revealed that electrocautery is a practical, well-tolerated outpatient procedure [28]. Side effects were minimal and short-term, with patients returning to baseline function within weeks. This supports the feasibility of integrating treatment into broader public health efforts.

### Implementation of anal cancer screening continuum: from screening to treatment

Given that anal cancer disproportionately affects high-risk populations, international and national guidelines now recommend screening for anal precancer through cytology and/or HPV testing [69]. Despite these recommendations, implementation and scale-up of screening practices remain limited due to a lack of systematic planning, infrastructure, availability of HRA and awareness.

A structured approach known as the anal screening cascade has been proposed to support the implementation of screening initiatives [70]. The cascade outlines each step from initial screening to confirmatory diagnosis via HRA, treatment, and long-term surveillance. By mapping this cascade, researchers and health systems can identify critical drop-off points and design interventions to address them. It also serves as a framework for formulating implementation research questions, such as which populations should be prioritised for screening, how often screening should occur, and how to manage follow-up care [70].

Studies from various settings highlight disparities in screening uptake and access to confirmatory services. For example, only 22% of people in HIV care in the United States received care at facilities with on-site HRA, and one-third attended clinics with no known access to HRA services [71]. Furthermore, there is evidence of inequities in treatment and follow-up. A study from the Mount Sinai Anal Dysplasia Programme in New York revealed that among individuals diagnosed with HSIL, only 25% received follow-up and 58% received treatment [72]. Race, insurance status, and HIV status were notable predictors of these outcomes.

Implementation science offers tools to address these gaps [73, 74]. By conducting needs assessments and identifying barriers and facilitators to implementation, tailored strategies can be selected using behavioural change frameworks such as the Theoretical Domains Framework [75]. For instance, preliminary qualitative research, performed prior to the release of the ANCHOR study findings, found that many healthcare providers lacked knowledge of updated screening guidelines and faced structural barriers such as limited HRA resources and competing priorities [76–83].

Barriers include stigma, lack of awareness and previous trauma; facilitators include education, self-collection options, peer support networks, clear clinical pathways, access to opinion leaders, and integration of screening into existing clinical workflows.

Ultimately, the success of anal cancer screening programmes hinges on systematic planning, evaluation of effectiveness and implementation outcomes, and a strong commitment to health equity. The anal screening cascade provides a valuable roadmap to identify where losses occur, allowing targeted improvements and sustainable scaling of services. Given that most healthcare settings are still in the early stages of adopting these practices, conducting a comprehensive needs assessment remains a crucial first step.

### Implementation of anal cancer screening: an evidence-based roadmap to successful implementation

Although HPV infection is the primary etiological agent for anal cancer, mirroring cervical carcinogenesis, there are notable biological and epidemiological differences. Anal HPV prevalence is high even in low-risk populations, and precancerous lesions are more common among HPV–positive high-risk women than in the cervix. However, progression from HSIL to invasive anal cancer occurs less frequently than in cervical disease, complicating the definition of actionable thresholds for intervention.

Current guidelines for anal cancer screening remain limited, with the Health and Human Services offering recommendations primarily for immunocompromised populations and the International Anal Neoplasia Society (IANS) (https://iansoc.org/Guidelines-and-Publications) for a broader range of populations at increased risk of anal cancer) [69]. The IANS guidelines, published in 2024, are the first systematic attempt to offer evidence-based screening protocols. They promote risk-based screening (category A, incidence more than tenfold higher) and shared decision-making (category B, incidence up to tenfold higher), emphasising that screening should be targeted to populations with sufficient incidence and healthcare infrastructure, particularly HRA capacity.

Screening methods include anal cytology, HPV testing, and the use of novel biomarkers such as p16/Ki-67 dual staining and HPV methylation [84]. Systematic reviews and meta-analyses have informed grading and recommendations for test use, although significant gaps remain. Specifically, data are lacking on the long-term performance of screening tests, the effectiveness of test combinations, and the applicability of biomarkers in different risk groups such as women living with HIV or HIV-negative GBMSM.

The IANS guidelines mark a significant milestone. Ongoing research priorities include longitudinal data collection, biomarker validation and establishing clinical risk thresholds. With focused effort, anal cancer prevention can follow the trajectory of cervical cancer elimination more efficiently and equitably.

## Advancing anal cancer screening: evidence-based approaches and real-world challenges

### Advancing anal cancer screening: experiences from Puerto Rico

Anal cancer, particularly SCC, has shown a rising incidence in Puerto Rico from 2000 to 2016, especially among PLWH. Research highlights a significantly increased standardised incidence ratio for secondary anal cancer in women with prior HPV-related gynaecologic neoplasms. In response, Puerto Rico has developed a targeted screening infrastructure, including the establishment of the Anal Neoplasia Clinic at the University of Puerto Rico Comprehensive Cancer Centre (UPRCCC). Since 2014, over 1,400 patients, of whom 73.8% PLWH, have been evaluated.

Recent studies identified knowledge gaps, barriers, and facilitators to anal cancer screening among PLWH and high-risk women. Factors such as patient trust in providers and integration of services in gynaecology clinics improved screening uptake. However, challenges remain, including a limited number of trained providers for HRA, inadequate referral systems, and low awareness of screening guidelines.

Ongoing studies are in progress under the CAMPO Consortium of the US NCI and aim to optimise screening algorithms. As HPV-related cancers continue to affect vulnerable populations disproportionately, expanding education, infrastructure and provider training are essential to ensure equitable access to screening and care.

### Advancing anal cancer screening: experiences from Belgium

Anal cancer screening in Belgium is gaining recognition as a critical public health need, particularly among high-risk populations. Based on current demographic estimates, over 9,500 PLWH aged over 50, more than 680 long-term transplant recipients, and up to 235,000 GBMSM and transwomen over 45 years old may require regular HRA. The increasing number of individuals in these categories underscores the urgent need to expand screening infrastructure and trained personnel.

Since 2015, Belgium has developed its HRA capacity through international training efforts and has adopted the IANS guidelines for standardisation. However, major bottlenecks remain, including insufficient coordination between medical specialties, limited availability of qualified HRA practitioners, and lack of standardised anal cytology procedures. Treatment options and reimbursement structures also require refinement to ensure sustainable implementation.

Despite these challenges, there is strong willingness among at-risk individuals to participate in screening programmes. Ensuring proper training, multidisciplinary coordination, and equitable access to screening and care will be essential to effectively address anal cancer prevention in Belgium.

### Real-world challenges in secondary anal cancer prevention: Discussion

Social and psychological factors significantly influence acceptance of anal cancer screening. Anal cancer is frequently associated with anal sex, which carries substantial stigma in many societies, discouraging individuals from seeking screening, despite epidemiological evidence showing that anal cancer is not necessarily linked to anal sexual practices.

Anticipated pain is another barrier. Anal self-sampling may mitigate embarrassment and increase privacy, but can introduce anxiety regarding the correct sampling technique.

Formal governmental guidelines and funding from national health programs and private insurance play an important role in normalising practice and providing medicolegal protection for healthcare providers. Clear policy frameworks facilitate routine implementation and professional confidence.

Beyond barriers to acceptance, structural barriers and constraints must also be addressed. The principal system-level limitations include limited access to HRA, insufficient clinician training, inadequate patient education, persistent stigma, and underdeveloped infrastructure. Given the limited HRA and treatment capacity, screening approaches must be optimised according to the prevalence of anal HSIL across different groups, and reliable biomarkers of progression and regression are needed to make efficient use of scarce HRA resources.

All current screening recommendations are based on expert opinion rather than prospective evidence. Critical gaps remain: when can we safely stop screening, what optimal follow-up algorithms after treatment should look like, and how to distinguish lesions likely to progress from those that will regress spontaneously.

In the ANCHOR study, progression from HSIL to cancer in the monitoring arm occurred at a rate of 400 per 100,000 person-years, and the risk of progression was 5.26-fold higher (95% CI 2.54–10.87) for lesions involving more than 50% of the anal/perianal canal compared with smaller lesions — providing an early basis for lesion-based risk stratification. Although timely treatment reduced anal cancer incidence by 57%, recurrence rates with hyfrecation remain high, underscoring the need for better therapeutic options. An optimal treatment would ideally be systemic or effective topically, HPV-specific, require no extended course, produce low recurrence rates, and carry a favourable safety profile — characteristics that would substantially reduce reliance on targeted HRA ablation and address multifocal disease more efficiently. Better treatment options and more efficient HRA training pathways must be pursued in parallel.

Clinician readiness is foundational; without trained providers and diagnostic capacity, awareness campaigns alone cannot translate into effective care. Parallel efforts to improve patient literacy, delivered in a destigmatised and culturally sensitive manner, further enhance uptake.

For high-risk groups, such as GBMSM and PLWH, individualised invitation systems demonstrate the highest effectiveness, though they are resource-intensive and difficult to operationalise in all settings. Integrating screening outreach into primary care services and collaborating with community-based organisations are critical strategies for reaching vulnerable populations and maintaining trust.

Screening algorithms significantly affect feasibility and scalability. More specific tests reduce unnecessary referrals for HRA, thereby alleviating demand on specialised services. Optimised triage pathways are essential to ensure that limited diagnostic capacity is reserved for individuals at highest risk. Efficient algorithms therefore represent a key determinant of sustainable program expansion.

## Lessons learned and way forward

### HPV primary prevention – expanding vaccination in male cohorts

A critical strategy towards the elimination of HPV-related disease, particularly in LMICs, is achieving high HPV vaccine coverage among girls. While the 90/70/90 targets remain the global benchmark, falling slightly short of these goals can still yield significant benefits. In settings where 80% vaccination or higher among girls is not feasible, universal vaccination with 70% coverage (including boys) may produce similar outcomes. Still, it is important to remember that vaccinating boys will not protect the hard-to-reach girls, as people often partner within their ethnic or social circles, where similar barriers to vaccination may exist.

Equity remains a persistent challenge, even in HICs, where access may be universal, but uptake is often uneven. This highlights the need to tailor interventions specifically to reach the most vulnerable populations, both in HICs and LMICs. These groups often experience barriers related to socio-economic status, education, and healthcare access, and require targeted outreach strategies.

For large countries with marked regional disparities, strategies targeting high-mortality or high-burden areas may generate greater immediate impact than uniform national scale-up. Geographically focused interventions can accelerate mortality reductions while broader system capacity is developed.

Sustained investment in communication and education is essential to maintaining public trust — particularly as demonstration projects succeed and complacency becomes a risk. Misinformation, whether spread through social media or amplified by respected community figures, remains a structural threat to programme sustainability. The Cameroon experience demonstrates that engaging religious and community stakeholders proactively — rather than reactively — is among the most effective tools available.

Changes in vaccine pricing by Gavi are expected in 2026, which may be favourable for more affordable HPV vaccines accessible to LMICs. This shift could be highly beneficial in improving vaccine coverage, especially where cost has been a key barrier.

Digital innovations such as digital consent, as piloted in Scotland, show promise in increasing participation rates by making processes more streamlined and accessible. These innovations should be explored more broadly to enhance equity and improve reach.

### HPV secondary prevention – non-cervical screening and program impact

Global estimates of HPV-related cancer burden are constrained by the uneven geographic distribution of population-based cancer registries, with coverage heavily concentrated in high-income countries. This discrepancy highlights significant underreporting in other regions, particularly in LMICs. Accurate data on HPV-related cancers beyond cervical cancer remain limited, especially in LMICs, where the burden is likely underestimated. Despite imperfections, the current data on HPV-associated cancers represent the best available evidence, and there is a pressing need to improve global surveillance and data collection to support effective policy and intervention strategies.

A critical limitation in anal cancer screening tools is the unknown negative predictive value, making it difficult to assess how well screening can reliably rule out disease. In addition, there is a strong need for the development of reliable biomarkers to better predict which lesions are likely to regress and which will progress, improving the effectiveness of treatment decisions. Although treatment following screening is shown to reduce disease progression by 57%, further enhancements are needed to increase this impact and reduce morbidity.

Self-sampling in anal cancer screening presents a promising avenue for expanding access and streamlining the screening cascade. Self-collected anal samples could serve as an efficient first step in screening, enabling broader reach while reserving high-resolution anoscopy (HRA) for those at highest risk of cancer progression. This approach could mirror developments in cervical cancer screening, where molecular triage tools such as methylation markers have demonstrated the capacity to screen, triage, and selectively refer — reducing unnecessary procedures without compromising detection. Studies in Puerto Rico have shown high concordance between self-collected and provider-collected anal samples. This suggests that self-sampling could expand access and alleviate workforce constraints. However, while some patients prefer provider-collected samples, it is crucial to educate and empower them to perform self-collection effectively and confidently. Continued investigation into the reliability, acceptability, and outcomes of self-sampling is essential to broadening screening reach.

Developing and implementing effective screening programmes require a multifaceted approach. As one presenter put it, providers need 'the guts to go to the anus' — a reminder that clinical confidence and institutional normalisation are prerequisites for screening uptake.

Still, human resource capacity remains a significant hurdle. Acquisition of competence requires substantial time on the part of the trainee and substantial trainer resources, underlining the importance of robust, hands-on training programmes. Beyond workforce training, comprehensive screening implementation must include quality assurance and control, real-world data collection, cost-effectiveness analyses by risk group, and studies on patient-reported outcomes to understand the impact on quality of life. Screening, HRA and treatment programs must be supported by adequate governmental or private insurance funding.

To remain relevant and responsive, a living guideline process for anal cancer screening is essential; one that evolves with emerging data and incorporates new testing technologies as they become available. Building sustainable capacity for screening, ensuring reimbursement mechanisms, and integrating ongoing research will be vital for making anal cancer screening a routine and impactful part of HPV-related cancer prevention.

Integrated 'see and treat' or one-stop-shop approaches — ensuring that screening leads directly to treatment within the same visit or pathway — are particularly relevant in settings where follow-up is unreliable. Experience-sharing across programmes, including frank discussion of what has not worked, accelerates implementation learning.

## Conclusion

HPV imposes a substantial burden of disease in both women and men, contributing not only to cervical cancer but also to anal, vulvar, vaginal, penile, and oropharyngeal malignancies. Men account for a significant proportion of HPV‑related cancers, and their lower seroconversion rates and sustained oral and genital HPV prevalence underscore the importance of direct protection. Evidence from millions of administered doses shows that HPV vaccines are safe, highly effective, and provide durable immunity in men and women alike. Population‑level benefits, including herd effects, further strengthen the case for broad vaccine deployment.

As the experience from the hepatitis B vaccination programme demonstrated, targeting only high‑risk groups is insufficient to reduce transmission at the population level. Similar limitations apply to HPV programmes that focus solely on adolescent girls — an approach that may inadvertently reinforce gendered hesitancy and limit educational reach. Excluding boys not only leaves them vulnerable but also perpetuates misconceptions that impede wider community uptake. Although vaccine supply is improving and single-dose schedules increase feasibility, resource constraints, particularly in LMICs, continue to challenge universal vaccination. In parallel, anal HPV infection and anal HSIL remain highly prevalent among GBMSM and immunocompromised populations, including PLWH and women with a history of other gynaecological cancers. Despite guideline endorsements for targeted anal cancer screening, implementation is inconsistent due to infrastructural, regulatory, and workforce limitations. Sustainable progress will require integrated planning, investment in training and quality assurance, and concerted efforts to engage underserved communities.

In summary, comprehensive prevention — combining vaccination, targeted screening, and equitable access — remains essential to reducing HPV-related disease globally, in both women and men.

## Data Availability

All the presentations of the meeting report are published on the website (www.hpvboard.org) after speakers’ approval.

## Abbreviations

AIN: Anal intraepithelial neoplasia
ART: Antiretroviral therapy
AS04: Aluminium hydroxide and 3-deacetylated monophosphoryl lipid A (adjuvant)
BLA: Biologics License Application
CI: Confidence interval
CIN3: Cervical intraepithelial neoplasia grade 3
COVID-19: Coronavirus disease 2019
DHS: Demographic and Health Surveys
GBMSM: Gay, bisexual, and other men who have sex with men
GDP: Gross domestic product
GLOBOCAN: Global Cancer Observatory database (IARC)
HBV: Hepatitis B virus
HIC: High-income country
HIV: Human immunodeficiency virus
HPV: Human papillomavirus
HRA: High-resolution anoscopy
HR-HPV: High-risk human papillomavirus
HSIL: High-grade squamous intraepithelial lesion
IANS: International Anal Neoplasia Society
IARC: International Agency for Research on Cancer
JCVI: Joint Committee on Vaccination and Immunisation
LMIC: Low- and middle-income country
NCI: (US) National Cancer Institute
NHS: National Health Service
NMPA: National Medical Products Administration (China)
NNV: Number needed to vaccinate
PIRI: Periodic intensification of routine immunisation
PLWH: People living with HIV
RRP: Recurrent respiratory papillomatosis
SCC: Squamous cell carcinoma
weaknesses,: SWOTStrengths,
and threats: Opportunities
TCA: Trichloroacetic acid
UPRCCC: University of Puerto Rico Comprehensive Cancer Centre
